# Redetermination and absolute configuration of (+)-7-epiclusianone

**DOI:** 10.1107/S1600536812043784

**Published:** 2012-10-27

**Authors:** Omar E. Christian, Frank R. Fronczek, Khoa Ky, Shreedu Pradhan, Anjela Manandhar, Cecilia Richmond

**Affiliations:** a Department of Chemistry, McNeese State University, Lake Charles, LA 70609, USA; b Department of Chemistry, Louisiana State University, Baton Rouge, LA 70803-1804, USA; c Louisiana Environmental Research Center, McNeese State University, Lake Charles, LA 70609, USA

## Abstract

The absolute configuration of 3-benzoyl-4-hy­droxy-6,6-dimethyl-1,5,7-tris­(3-methyl­but-2-en­yl)bicyclo­[3.3.1]non-3-ene-2,9-dione, C_33_H_42_O_4_, isolated from *Hypericum hypericoides*, has been determined. The previous study [Xiao *et al.* (2007 ▶). *J. Nat. Prod.* **70**, 1779–1782] gave only the established relative configuration. The three stereogenic centers are now established as 1*R*, 5*R* and 7*S* on the basis of the refinement of the Flack absolute structure parameter against Cu *K*α data and correspond to a specific rotation of [α]_*D*_^20^ = +66°. The enol–hy­droxy group forms an intra­molecular O—H⋯O hydrogen bond to close an *S*(6) ring.

## Related literature

For a review of polycyclic polyprenylated acyl­phloroglucinols, see: Ciochina & Grossman (2006 ▶). For background to Clusiaceae metabolites, see: Garnsey *et al.* (2011 ▶); Zhang *et al.* (2010 ▶); Christian *et al.* (2008 ▶); Wu *et al.* (2008 ▶). For relative-configuration structure determinatons, see: Santos *et al.* (1998 ▶); Xiao *et al.* (2007 ▶); Martins *et al.* (2009 ▶). For related structures, see: McCandlish *et al.* (1976 ▶); Fronczek *et al.* (2012 ▶). For optical rotation results for the title compound, see: Piccinelli *et al.* (2005 ▶) and for related compounds, see: Tanaka *et al.* (2004 ▶). For keto–enol tautomerism in related compounds, see: Martins *et al.* (2007 ▶). For absolute configuration based on resonant scattering from light atoms, see: Hooft *et al.* (2008 ▶)
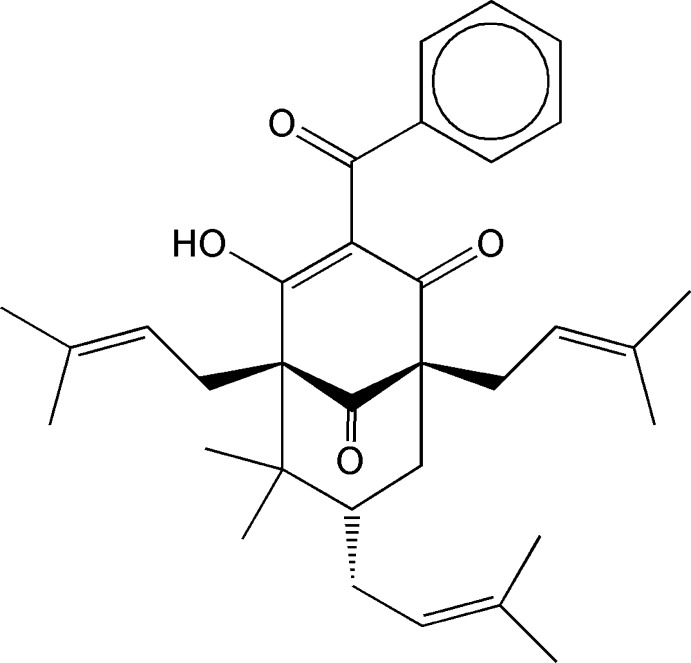


## Experimental

### 

#### Crystal data

C_33_H_42_O_4_
*M* *_r_* = 502.67
Orthorhombic, 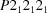

*a* = 8.6177 (4) Å
*b* = 12.4157 (6) Å
*c* = 26.8632 (13) Å
*V* = 2874.2 (2) Å^3^
*Z* = 4
Cu *K*α radiation
μ = 0.59 mm^−1^
*T* = 90 K
0.25 × 0.24 × 0.16 mm

#### Data collection

Bruker Kappa APEXII CCD DUO diffractometer
Absorption correction: multi-scan (*SADABS*; Sheldrick, 2004 ▶) *T*_min_ = 0.824, *T*_max_ = 0.889
17192 measured reflections
5171 independent reflections
5131 reflections with *I* > 2σ(*I*)
*R*_int_ = 0.029

#### Refinement

*R*[*F* ^2^ > 2σ(*F* ^2^)] = 0.028
*wR*(*F* ^2^) = 0.074
*S* = 1.03
5171 reflections
345 parameters
H atoms treated by a mixture of independent and constrained refinement
Δρ_max_ = 0.22 e Å^−3^
Δρ_min_ = −0.14 e Å^−3^
Absolute structure: Flack (1983 ▶), 2200 Friedel pairs
Flack parameter: 0.04 (12)

### 

Data collection: *APEX2* (Bruker, 2006 ▶); cell refinement: *SAINT* (Bruker, 2006 ▶); data reduction: *SAINT*; program(s) used to solve structure: *SHELXS97* (Sheldrick, 2008 ▶); program(s) used to refine structure: *SHELXL97* (Sheldrick, 2008 ▶); molecular graphics: *ORTEP-3 for Windows* (Farrugia, 1997 ▶); software used to prepare material for publication: *SHELXTL* (Sheldrick, 2008 ▶).

## Supplementary Material

Crystal structure: contains datablock(s) global, I. DOI: 10.1107/S1600536812043784/hb6978sup1.cif

Structure factors: contains datablock(s) I. DOI: 10.1107/S1600536812043784/hb6978Isup2.hkl

Supplementary material file. DOI: 10.1107/S1600536812043784/hb6978Isup3.cml

Additional supplementary materials:  crystallographic information; 3D view; checkCIF report

## Figures and Tables

**Table 1:** Hydrogen-bond geometry (Å, °)

*D*—H⋯*A*	*D*—H	H⋯*A*	*D*⋯*A*	*D*—H⋯*A*
O2—H2*O*⋯O4	1.014 (16)	1.477 (16)	2.4368 (12)	155.7 (15)

**Fig. 1.**
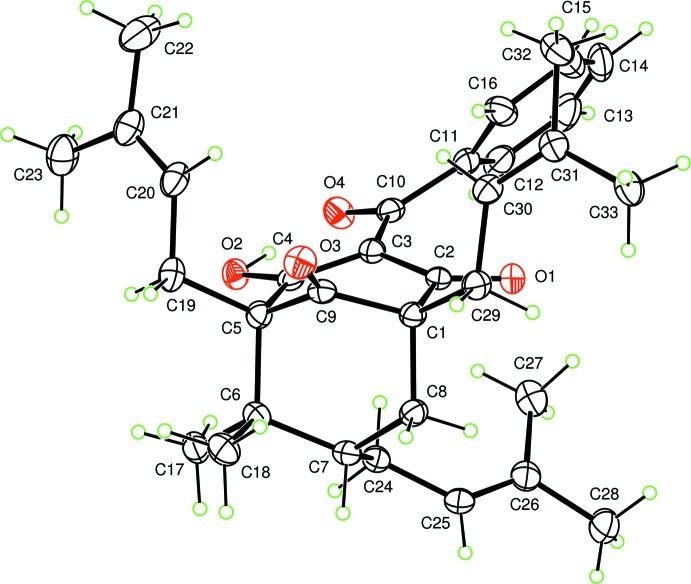
Ellipsoids at the 50% level, with H atoms having arbitrary radius.

**Fig. 2.**
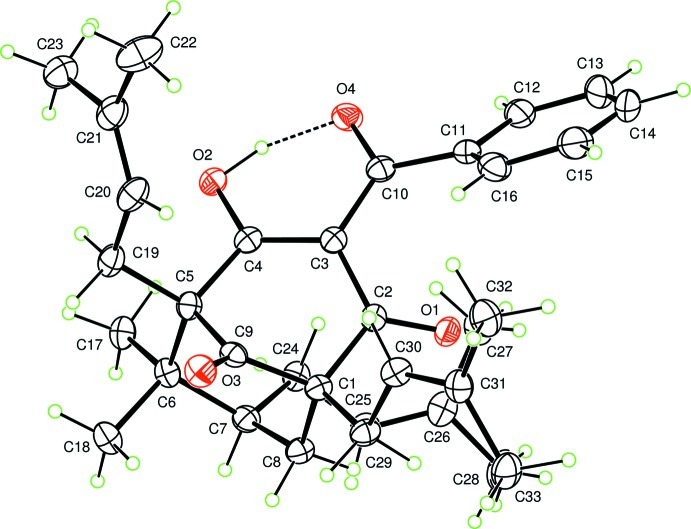
View of the molecule showing the intramolecular hydrogen bond; 50% ellipsoids

## supplementary crystallographic information

### Comment

Recently, there has been a resurgence of interest in the Clusiaceae family due mainly to the significant synthetic challenge presented by several compound classes isolated from this family, in particular the benzophenone-type metabolites which contain the bridged bicyclics (Garnsey *et al.*, 2011) and tricyclic cores (Zhang *et al.*, 2010). The simpler and stereochemically less dense bicyclononanes occur frequently in the genus *Hypericum* (Christian *et al.*, 2008), plants related to St. John's Wort (Ciochina & Grossman, 2006). The *Hypericum* genus is one of largest in the Clusiaceae family of plants and is distributed worldwide. The phloroglucinol derived metabolites from various species within this genus have shown good potential as antioxidants (Wu *et al.*, 2008). The hexane extract of *Hypericum hypericoides* collected in Lake Charles, Louisiana, yielded the title compound (I). 7-Epiclusianone (I) has previously been isolated from *Rheedia gardneriana* (Santos *et al.*, 1998), *H. sampsonii* (Xiao *et al.*, 2007) and from a Jamaican collection of *H. hypericoides* (Christian *et al.*, 2008). The gross structure was confirmed by ^1^H NMR, ^13^C NMR and DEPT analysis displaying the diagnostic resonances for the bicyclononane core, in addition to the requisite ^3^*J*_HH_ coupling to establish the C-7 prenyl group as axial (Piccinelli *et al.*, 2005, Christian *et al.*, 2008). The melting point (365–366 K) and specific rotation (+66°) of (I) was similar to that of Santos *et al.*, (1998) (+77°), Piccinelli *et al.*, (2005) (+62.3°) and Christian *et al.*, (2008) (+67.5°). However, these values are in stark contrast to the *H. sampsonii* collection isolated by Xiao and coworkers, which gave a specific rotation of -9.65° (Xiao *et al.*, 2007). This ambiguity in the stereochemistry and a lack of absolute structural data prompted this investigation to unequivocally determine the absolute configuration of (I) and correlate it with chiroptical data.

The structure of (I) has been reported several times, all at room temperature and yielding only relative configuration (Santos *et al.*, 1998; Xiao *et al.*, 2007; Martins *et al.*, 2009). Our low-temperature Cu *K*α data with 2200 Bijvoet pairs allowed unambiguous determination of the absolute configuration from the Flack (1983) parameter *x*=0.04 (12). The Hooft *et al.*, (2008) analysis yielded *y*=0.02 (4) and P2(true)=1.000. This configuration is depicted in Fig. 1, and has the *R* configuration at C1 and C5, and the *S* configuration at C7.

Keto-enol tautomerism is a common feature in natural polyprenylated benzophenones (Martins *et al.*, 2007), and also exists in (I). In the solid, the C═C double bond is between C3 and C4, with distance 1.3932 (16) Å, and C2═O1 is a ketone, with distance 1.2169 (13) Å. Hydroxy group O2 forms an intramolecular hydrogen bond to the benzophenone O4, as shown in Fig. 2.

The quite different [α]_D_^20^ value of -9.65° for 7-epiclusianone from *H. sampsonii* reported by Xiao *et al.*, (2007) is of considerable interest, particularly since that structure was confirmed by crystal structure determination. It seems likely that their sample was a partial racemate. Closely related polyprenylated phloroglucinols have been found to be racemic by crystal structure determination. Clusianone from *Clusia congestiflora* crystallizes in racemic Pna2_1_ (McCandlish *et al.*, 1976). Hyperibone *L* from *H. dolabriforme* differs from (I) only by having a methyl group instead of an prenyl group at C5, and crystallizes in racemic P-1 (Fronczek *et al.*, 2012), while it has been reported with an optical rotation of +69.5° from *H. scabrum* (Tanaka *et al.*, 2004). Since no obvious means of racemization of these compounds during isolation and crystallization is apparent, the plants appear to commonly produce both enantiomers, and in unequal amounts.

### Experimental

*Hypericum hypericoides* was collected from Sam Houston Jones State Park, Calcasieu Parish, LA, (N 30 18.1246, W 93 15.5163) in June 2011. Voucher specimens are preserved in the Herbarium, Department of Biological Sciences, McNeese State University. The dried and pulverized roots of *H. hypericoides* (204 g) were extracted with hexane (3 *x* 1.5 *L*). The evaporation of the hexanes yielded a yellowish gum (4.3 g). The hexane extract was chromatographed over silica gel and eluted with 0 – 100% hexane/ethyl acetate mixtures to yield fifteen fractions (B1 – B15). Fraction B2 yielded 7-epiclusianone (I), and slow evaporation from methanol yielded suitable crystals. The crystals were colorless and cube-like (m.p. 365 - 366 K). The HREIMS, ^1^H and ^13^C NMR were the same as indicated in the literature (Christian *et al.*, 2008).

### Refinement

H atoms on C were placed in idealized positions with C—H distances 0.95 - 1.00 Å and thereafter treated as riding. Coordinates of the OH hydrogen atom were refined. A torional parameter was refined for each methyl group. *U*_iso_ for H were assigned as 1.2 times *U*_eq_ of the attached atoms (1.5 for methyl and OH).

### Crystal data

**Table:**

C_33_H_42_O_4_	*F*(000) = 1088
*M**_r_* = 502.67	*D*_x_ = 1.162 Mg m^−^^3^
Orthorhombic, *P*2_1_2_1_2_1_	Cu *K*α radiation, λ = 1.54184 Å
Hall symbol: P 2ac 2ab	Cell parameters from 9866 reflections
*a* = 8.6177 (4) Å	θ = 6.1–68.7°
*b* = 12.4157 (6) Å	µ = 0.59 mm^−^^1^
*c* = 26.8632 (13) Å	*T* = 90 K
*V* = 2874.2 (2) Å^3^	Fragment, colourless
*Z* = 4	0.25 × 0.24 × 0.16 mm

### Data collection

**Table:**

Bruker Kappa APEXII CCD DUO diffractometer	5171 independent reflections
Radiation source: IµS microfocus	5131 reflections with *I* > 2σ(*I*)
QUAZAR multilayer optics monochromator	*R*_int_ = 0.029
φ and ω scans	θ_max_ = 69.0°, θ_min_ = 6.1°
Absorption correction: multi-scan (*SADABS*; Sheldrick, 2004)	*h* = −7→10
*T*_min_ = 0.824, *T*_max_ = 0.889	*k* = −14→14
17192 measured reflections	*l* = −26→32

### Refinement

**Table:**

Refinement on *F*^2^	Secondary atom site location: difference Fourier map
Least-squares matrix: full	Hydrogen site location: inferred from neighbouring sites
*R*[*F*^2^ > 2σ(*F*^2^)] = 0.028	H atoms treated by a mixture of independent and constrained refinement
*wR*(*F*^2^) = 0.074	*w* = 1/[σ^2^(*F*_o_^2^) + (0.0421*P*)^2^ + 0.5041*P*] where *P* = (*F*_o_^2^ + 2*F*_c_^2^)/3
*S* = 1.03	(Δ/σ)_max_ = 0.001
5171 reflections	Δρ_max_ = 0.22 e Å^−^^3^
345 parameters	Δρ_min_ = −0.14 e Å^−^^3^
0 restraints	Absolute structure: Flack (1983), 2200 Friedel pairs
Primary atom site location: structure-invariant direct methods	Flack parameter: 0.04 (12)

### Special details

**Table:**

Geometry. All e.s.d.'s (except the e.s.d. in the dihedral angle between two l.s. planes) are estimated using the full covariance matrix. The cell e.s.d.'s are taken into account individually in the estimation of e.s.d.'s in distances, angles and torsion angles; correlations between e.s.d.'s in cell parameters are only used when they are defined by crystal symmetry. An approximate (isotropic) treatment of cell e.s.d.'s is used for estimating e.s.d.'s involving l.s. planes.
Refinement. Refinement of *F*^2^ against ALL reflections. The weighted *R*-factor *wR* and goodness of fit *S* are based on *F*^2^, conventional *R*-factors *R* are based on *F*, with *F* set to zero for negative *F*^2^. The threshold expression of *F*^2^ > σ(*F*^2^) is used only for calculating *R*-factors(gt) *etc*. and is not relevant to the choice of reflections for refinement. *R*-factors based on *F*^2^ are statistically about twice as large as those based on *F*, and *R*- factors based on ALL data will be even larger.

### Fractional atomic coordinates and isotropic or equivalent isotropic displacement parameters (Å^2^)

**Table:**

	*x*	*y*	*z*	*U*_iso_*/*U*_eq_	
O1	0.38204 (9)	0.57722 (6)	0.55937 (3)	0.02174 (17)	
O2	0.19699 (9)	0.30231 (6)	0.66019 (3)	0.02165 (18)	
H2O	0.1128 (18)	0.3151 (13)	0.6345 (6)	0.032*	
O3	0.55907 (10)	0.53739 (7)	0.71815 (3)	0.02443 (18)	
O4	0.02182 (10)	0.37865 (7)	0.59833 (3)	0.02514 (19)	
C1	0.53797 (13)	0.53245 (9)	0.62906 (4)	0.0180 (2)	
C2	0.38730 (13)	0.52889 (8)	0.59874 (4)	0.0171 (2)	
C3	0.26005 (13)	0.46092 (9)	0.61697 (4)	0.0180 (2)	
C4	0.29307 (13)	0.38075 (9)	0.65168 (4)	0.0177 (2)	
C5	0.43928 (13)	0.37919 (9)	0.68338 (4)	0.0190 (2)	
C6	0.56654 (13)	0.29794 (9)	0.66112 (4)	0.0209 (2)	
C7	0.61814 (14)	0.33294 (9)	0.60776 (4)	0.0204 (2)	
H7	0.7201	0.2961	0.6026	0.024*	
C8	0.65687 (13)	0.45420 (9)	0.60513 (4)	0.0205 (2)	
H8A	0.6689	0.4742	0.5697	0.025*	
H8B	0.7585	0.4656	0.6215	0.025*	
C9	0.51344 (13)	0.49077 (9)	0.68128 (4)	0.0184 (2)	
C10	0.10833 (13)	0.45927 (9)	0.59317 (4)	0.0197 (2)	
C11	0.04524 (13)	0.55219 (10)	0.56511 (4)	0.0218 (2)	
C12	−0.05267 (14)	0.53185 (12)	0.52473 (5)	0.0291 (3)	
H12	−0.0719	0.4599	0.5144	0.035*	
C13	−0.12146 (17)	0.61707 (14)	0.49996 (5)	0.0397 (4)	
H13	−0.1865	0.6034	0.4721	0.048*	
C14	−0.09644 (17)	0.72184 (14)	0.51530 (6)	0.0431 (4)	
H14	−0.1446	0.7799	0.4982	0.052*	
C15	−0.00110 (17)	0.74202 (12)	0.55568 (6)	0.0399 (3)	
H15	0.0150	0.8140	0.5665	0.048*	
C16	0.07115 (15)	0.65772 (10)	0.58051 (5)	0.0288 (3)	
H16	0.1379	0.6720	0.6079	0.035*	
C17	0.50800 (15)	0.18136 (9)	0.66151 (4)	0.0245 (3)	
H17A	0.5839	0.1349	0.6449	0.037*	
H17B	0.4085	0.1773	0.6439	0.037*	
H17C	0.4942	0.1574	0.6960	0.037*	
C18	0.71138 (14)	0.30162 (11)	0.69510 (5)	0.0268 (3)	
H18A	0.6842	0.2745	0.7282	0.040*	
H18B	0.7482	0.3761	0.6978	0.040*	
H18C	0.7934	0.2566	0.6808	0.040*	
C19	0.39058 (15)	0.35180 (10)	0.73731 (4)	0.0241 (3)	
H19A	0.3508	0.2770	0.7384	0.029*	
H19B	0.4828	0.3557	0.7592	0.029*	
C20	0.26812 (16)	0.42675 (11)	0.75676 (4)	0.0268 (3)	
H20	0.2918	0.5014	0.7552	0.032*	
C21	0.13033 (16)	0.40148 (11)	0.77591 (4)	0.0282 (3)	
C22	0.02230 (18)	0.48786 (12)	0.79411 (6)	0.0406 (3)	
H22A	0.0692	0.5587	0.7883	0.061*	
H22B	0.0035	0.4781	0.8298	0.061*	
H22C	−0.0763	0.4831	0.7761	0.061*	
C23	0.07067 (16)	0.28880 (12)	0.78242 (5)	0.0350 (3)	
H23A	0.1448	0.2378	0.7680	0.052*	
H23B	−0.0297	0.2816	0.7656	0.052*	
H23C	0.0579	0.2734	0.8180	0.052*	
C24	0.51574 (14)	0.29320 (9)	0.56401 (4)	0.0210 (2)	
H24A	0.5060	0.2138	0.5656	0.025*	
H24B	0.4105	0.3245	0.5672	0.025*	
C25	0.58384 (15)	0.32466 (9)	0.51460 (4)	0.0241 (3)	
H25	0.6906	0.3082	0.5102	0.029*	
C26	0.51463 (16)	0.37244 (9)	0.47629 (4)	0.0251 (3)	
C27	0.34689 (16)	0.40573 (10)	0.47428 (5)	0.0288 (3)	
H27A	0.2939	0.3815	0.5046	0.043*	
H27B	0.2974	0.3730	0.4451	0.043*	
H27C	0.3401	0.4843	0.4718	0.043*	
C28	0.60348 (19)	0.39918 (11)	0.42989 (5)	0.0356 (3)	
H28A	0.7113	0.3754	0.4336	0.053*	
H28B	0.6010	0.4772	0.4244	0.053*	
H28C	0.5562	0.3624	0.4014	0.053*	
C29	0.60306 (14)	0.64717 (9)	0.62838 (4)	0.0211 (2)	
H29A	0.6948	0.6507	0.6505	0.025*	
H29B	0.6378	0.6647	0.5942	0.025*	
C30	0.48630 (14)	0.72998 (9)	0.64493 (4)	0.0213 (2)	
H30	0.4012	0.7045	0.6641	0.026*	
C31	0.48995 (14)	0.83484 (9)	0.63557 (4)	0.0218 (2)	
C32	0.36423 (15)	0.90856 (10)	0.65404 (5)	0.0280 (3)	
H32A	0.2827	0.8659	0.6701	0.042*	
H32B	0.3199	0.9483	0.6259	0.042*	
H32C	0.4080	0.9595	0.6781	0.042*	
C33	0.62042 (16)	0.88899 (10)	0.60776 (5)	0.0275 (3)	
H33A	0.6857	0.9288	0.6313	0.041*	
H33B	0.5772	0.9389	0.5831	0.041*	
H33C	0.6831	0.8344	0.5908	0.041*	

### Atomic displacement parameters (Å^2^)

**Table:**

	*U*^11^	*U*^22^	*U*^33^	*U*^12^	*U*^13^	*U*^23^
O1	0.0240 (4)	0.0205 (4)	0.0207 (4)	−0.0005 (3)	0.0017 (3)	0.0040 (3)
O2	0.0212 (4)	0.0193 (4)	0.0244 (4)	−0.0034 (3)	0.0001 (3)	0.0034 (3)
O3	0.0267 (4)	0.0254 (4)	0.0211 (4)	−0.0026 (3)	−0.0026 (3)	−0.0038 (3)
O4	0.0198 (4)	0.0214 (4)	0.0342 (5)	−0.0033 (3)	−0.0018 (3)	0.0030 (4)
C1	0.0181 (5)	0.0161 (5)	0.0198 (5)	0.0000 (4)	0.0021 (4)	−0.0011 (4)
C2	0.0205 (5)	0.0139 (5)	0.0170 (5)	0.0028 (4)	0.0026 (4)	−0.0016 (4)
C3	0.0190 (5)	0.0169 (5)	0.0181 (5)	0.0008 (4)	0.0013 (4)	−0.0013 (4)
C4	0.0200 (5)	0.0158 (5)	0.0173 (5)	0.0002 (4)	0.0037 (4)	−0.0032 (4)
C5	0.0213 (5)	0.0193 (5)	0.0165 (5)	0.0002 (5)	−0.0002 (4)	0.0002 (4)
C6	0.0222 (5)	0.0195 (6)	0.0212 (6)	0.0024 (4)	−0.0023 (4)	0.0013 (5)
C7	0.0197 (5)	0.0187 (5)	0.0227 (6)	0.0034 (5)	0.0019 (5)	−0.0009 (4)
C8	0.0191 (5)	0.0210 (6)	0.0214 (5)	−0.0003 (4)	0.0028 (4)	−0.0003 (5)
C9	0.0156 (5)	0.0196 (5)	0.0198 (5)	0.0027 (4)	0.0003 (4)	−0.0015 (4)
C10	0.0195 (5)	0.0192 (5)	0.0203 (5)	0.0009 (4)	0.0037 (4)	−0.0025 (4)
C11	0.0163 (5)	0.0257 (6)	0.0233 (5)	0.0022 (4)	0.0039 (4)	0.0043 (5)
C12	0.0228 (6)	0.0431 (7)	0.0215 (6)	0.0035 (6)	0.0029 (5)	0.0037 (6)
C13	0.0300 (7)	0.0636 (10)	0.0256 (6)	0.0093 (7)	0.0022 (6)	0.0168 (7)
C14	0.0304 (7)	0.0518 (9)	0.0472 (9)	0.0132 (7)	0.0101 (6)	0.0325 (7)
C15	0.0291 (7)	0.0271 (7)	0.0635 (10)	0.0057 (5)	0.0101 (7)	0.0162 (7)
C16	0.0216 (6)	0.0248 (6)	0.0400 (7)	0.0033 (5)	0.0026 (5)	0.0040 (5)
C17	0.0304 (6)	0.0203 (6)	0.0229 (6)	0.0016 (5)	−0.0007 (5)	0.0033 (5)
C18	0.0252 (6)	0.0275 (6)	0.0276 (6)	0.0040 (5)	−0.0057 (5)	0.0025 (5)
C19	0.0296 (6)	0.0254 (6)	0.0173 (5)	−0.0037 (5)	−0.0003 (5)	0.0027 (4)
C20	0.0349 (7)	0.0270 (6)	0.0187 (5)	−0.0057 (5)	0.0047 (5)	−0.0014 (5)
C21	0.0327 (7)	0.0335 (7)	0.0183 (5)	−0.0045 (6)	0.0007 (5)	−0.0006 (5)
C22	0.0412 (8)	0.0408 (8)	0.0398 (8)	−0.0073 (7)	0.0164 (6)	−0.0087 (6)
C23	0.0298 (7)	0.0398 (8)	0.0352 (7)	−0.0082 (6)	0.0003 (6)	0.0082 (6)
C24	0.0255 (6)	0.0166 (5)	0.0210 (5)	0.0017 (4)	0.0018 (5)	−0.0013 (4)
C25	0.0290 (6)	0.0191 (6)	0.0241 (6)	0.0045 (5)	0.0052 (5)	−0.0028 (5)
C26	0.0364 (7)	0.0168 (5)	0.0222 (6)	−0.0001 (5)	0.0029 (5)	−0.0041 (5)
C27	0.0358 (7)	0.0252 (6)	0.0253 (6)	−0.0022 (5)	−0.0072 (5)	−0.0009 (5)
C28	0.0514 (9)	0.0308 (7)	0.0245 (6)	0.0054 (6)	0.0077 (6)	0.0029 (5)
C29	0.0206 (6)	0.0185 (6)	0.0242 (6)	−0.0031 (4)	0.0021 (5)	−0.0006 (4)
C30	0.0226 (6)	0.0211 (6)	0.0202 (5)	−0.0031 (5)	0.0012 (4)	−0.0032 (4)
C31	0.0267 (6)	0.0209 (5)	0.0177 (5)	−0.0014 (5)	−0.0042 (5)	−0.0019 (4)
C32	0.0327 (7)	0.0215 (6)	0.0297 (6)	0.0034 (5)	−0.0037 (5)	−0.0008 (5)
C33	0.0338 (7)	0.0187 (6)	0.0300 (6)	−0.0027 (5)	−0.0014 (5)	0.0031 (5)

### Geometric parameters (Å, º)

**Table:**

O1—C2	1.2169 (13)	C18—H18B	0.9800
O2—C4	1.2986 (14)	C18—H18C	0.9800
O2—H2O	1.014 (16)	C19—C20	1.5009 (18)
O3—C9	1.2126 (14)	C19—H19A	0.9900
O4—C10	1.2558 (15)	C19—H19B	0.9900
O4—H2O	1.477 (16)	C20—C21	1.3316 (19)
C1—C9	1.5103 (15)	C20—H20	0.9500
C1—C29	1.5309 (15)	C21—C23	1.5007 (19)
C1—C2	1.5333 (15)	C21—C22	1.5019 (19)
C1—C8	1.5515 (15)	C22—H22A	0.9800
C2—C3	1.4679 (15)	C22—H22B	0.9800
C3—C4	1.3932 (16)	C22—H22C	0.9800
C3—C10	1.4556 (16)	C23—H23A	0.9800
C4—C5	1.5208 (16)	C23—H23B	0.9800
C5—C9	1.5267 (16)	C23—H23C	0.9800
C5—C19	1.5464 (15)	C24—C25	1.5028 (16)
C5—C6	1.6055 (16)	C24—H24A	0.9900
C6—C17	1.5329 (16)	C24—H24B	0.9900
C6—C18	1.5471 (16)	C25—C26	1.3291 (18)
C6—C7	1.5624 (16)	C25—H25	0.9500
C7—C8	1.5437 (16)	C26—C28	1.5002 (18)
C7—C24	1.5504 (16)	C26—C27	1.5044 (19)
C7—H7	1.0000	C27—H27A	0.9800
C8—H8A	0.9900	C27—H27B	0.9800
C8—H8B	0.9900	C27—H27C	0.9800
C10—C11	1.4815 (16)	C28—H28A	0.9800
C11—C16	1.3920 (18)	C28—H28B	0.9800
C11—C12	1.3972 (18)	C28—H28C	0.9800
C12—C13	1.383 (2)	C29—C30	1.5057 (16)
C12—H12	0.9500	C29—H29A	0.9900
C13—C14	1.381 (3)	C29—H29B	0.9900
C13—H13	0.9500	C30—C31	1.3264 (17)
C14—C15	1.384 (2)	C30—H30	0.9500
C14—H14	0.9500	C31—C32	1.5025 (17)
C15—C16	1.3886 (19)	C31—C33	1.5080 (17)
C15—H15	0.9500	C32—H32A	0.9800
C16—H16	0.9500	C32—H32B	0.9800
C17—H17A	0.9800	C32—H32C	0.9800
C17—H17B	0.9800	C33—H33A	0.9800
C17—H17C	0.9800	C33—H33B	0.9800
C18—H18A	0.9800	C33—H33C	0.9800
			
C4—O2—H2O	102.7 (9)	H18A—C18—H18C	109.5
C10—O4—H2O	100.6 (6)	H18B—C18—H18C	109.5
C9—C1—C29	112.40 (9)	C20—C19—C5	112.37 (10)
C9—C1—C2	111.41 (9)	C20—C19—H19A	109.1
C29—C1—C2	109.32 (9)	C5—C19—H19A	109.1
C9—C1—C8	105.23 (9)	C20—C19—H19B	109.1
C29—C1—C8	109.62 (9)	C5—C19—H19B	109.1
C2—C1—C8	108.74 (9)	H19A—C19—H19B	107.9
O1—C2—C3	123.07 (10)	C21—C20—C19	127.98 (12)
O1—C2—C1	118.62 (10)	C21—C20—H20	116.0
C3—C2—C1	118.16 (9)	C19—C20—H20	116.0
C4—C3—C10	117.87 (10)	C20—C21—C23	124.76 (13)
C4—C3—C2	118.78 (10)	C20—C21—C22	120.69 (12)
C10—C3—C2	122.18 (10)	C23—C21—C22	114.54 (12)
O2—C4—C3	121.56 (10)	C21—C22—H22A	109.5
O2—C4—C5	114.85 (10)	C21—C22—H22B	109.5
C3—C4—C5	123.57 (10)	H22A—C22—H22B	109.5
C4—C5—C9	108.34 (9)	C21—C22—H22C	109.5
C4—C5—C19	107.61 (9)	H22A—C22—H22C	109.5
C9—C5—C19	110.34 (9)	H22B—C22—H22C	109.5
C4—C5—C6	111.44 (9)	C21—C23—H23A	109.5
C9—C5—C6	105.69 (9)	C21—C23—H23B	109.5
C19—C5—C6	113.34 (9)	H23A—C23—H23B	109.5
C17—C6—C18	106.82 (10)	C21—C23—H23C	109.5
C17—C6—C7	111.26 (9)	H23A—C23—H23C	109.5
C18—C6—C7	107.67 (9)	H23B—C23—H23C	109.5
C17—C6—C5	111.47 (9)	C25—C24—C7	111.38 (10)
C18—C6—C5	108.23 (9)	C25—C24—H24A	109.4
C7—C6—C5	111.18 (9)	C7—C24—H24A	109.4
C8—C7—C24	113.50 (9)	C25—C24—H24B	109.4
C8—C7—C6	112.01 (9)	C7—C24—H24B	109.4
C24—C7—C6	116.42 (9)	H24A—C24—H24B	108.0
C8—C7—H7	104.5	C26—C25—C24	128.65 (12)
C24—C7—H7	104.5	C26—C25—H25	115.7
C6—C7—H7	104.5	C24—C25—H25	115.7
C7—C8—C1	116.67 (9)	C25—C26—C28	120.87 (13)
C7—C8—H8A	108.1	C25—C26—C27	125.57 (12)
C1—C8—H8A	108.1	C28—C26—C27	113.56 (11)
C7—C8—H8B	108.1	C26—C27—H27A	109.5
C1—C8—H8B	108.1	C26—C27—H27B	109.5
H8A—C8—H8B	107.3	H27A—C27—H27B	109.5
O3—C9—C1	123.35 (10)	C26—C27—H27C	109.5
O3—C9—C5	122.61 (10)	H27A—C27—H27C	109.5
C1—C9—C5	113.81 (9)	H27B—C27—H27C	109.5
O4—C10—C3	119.73 (10)	C26—C28—H28A	109.5
O4—C10—C11	117.33 (10)	C26—C28—H28B	109.5
C3—C10—C11	122.84 (10)	H28A—C28—H28B	109.5
C16—C11—C12	119.85 (12)	C26—C28—H28C	109.5
C16—C11—C10	121.52 (11)	H28A—C28—H28C	109.5
C12—C11—C10	118.42 (11)	H28B—C28—H28C	109.5
C13—C12—C11	119.60 (14)	C30—C29—C1	112.76 (9)
C13—C12—H12	120.2	C30—C29—H29A	109.0
C11—C12—H12	120.2	C1—C29—H29A	109.0
C14—C13—C12	120.65 (14)	C30—C29—H29B	109.0
C14—C13—H13	119.7	C1—C29—H29B	109.0
C12—C13—H13	119.7	H29A—C29—H29B	107.8
C13—C14—C15	119.81 (13)	C31—C30—C29	126.76 (11)
C13—C14—H14	120.1	C31—C30—H30	116.6
C15—C14—H14	120.1	C29—C30—H30	116.6
C14—C15—C16	120.41 (15)	C30—C31—C32	121.22 (12)
C14—C15—H15	119.8	C30—C31—C33	123.31 (11)
C16—C15—H15	119.8	C32—C31—C33	115.44 (10)
C15—C16—C11	119.66 (13)	C31—C32—H32A	109.5
C15—C16—H16	120.2	C31—C32—H32B	109.5
C11—C16—H16	120.2	H32A—C32—H32B	109.5
C6—C17—H17A	109.5	C31—C32—H32C	109.5
C6—C17—H17B	109.5	H32A—C32—H32C	109.5
H17A—C17—H17B	109.5	H32B—C32—H32C	109.5
C6—C17—H17C	109.5	C31—C33—H33A	109.5
H17A—C17—H17C	109.5	C31—C33—H33B	109.5
H17B—C17—H17C	109.5	H33A—C33—H33B	109.5
C6—C18—H18A	109.5	C31—C33—H33C	109.5
C6—C18—H18B	109.5	H33A—C33—H33C	109.5
H18A—C18—H18B	109.5	H33B—C33—H33C	109.5
C6—C18—H18C	109.5		
			
C9—C1—C2—O1	167.02 (10)	C29—C1—C9—C5	176.44 (9)
C29—C1—C2—O1	42.19 (13)	C2—C1—C9—C5	53.35 (12)
C8—C1—C2—O1	−77.45 (12)	C8—C1—C9—C5	−64.32 (11)
C9—C1—C2—C3	−17.26 (14)	C4—C5—C9—O3	133.48 (11)
C29—C1—C2—C3	−142.09 (10)	C19—C5—C9—O3	15.92 (15)
C8—C1—C2—C3	98.26 (11)	C6—C5—C9—O3	−106.98 (12)
O1—C2—C3—C4	157.56 (11)	C4—C5—C9—C1	−51.93 (12)
C1—C2—C3—C4	−17.96 (15)	C19—C5—C9—C1	−169.49 (9)
O1—C2—C3—C10	−9.79 (17)	C6—C5—C9—C1	67.62 (11)
C1—C2—C3—C10	174.70 (9)	C4—C3—C10—O4	−10.85 (16)
C10—C3—C4—O2	4.87 (16)	C2—C3—C10—O4	156.60 (11)
C2—C3—C4—O2	−163.02 (10)	C4—C3—C10—C11	165.27 (11)
C10—C3—C4—C5	−173.26 (10)	C2—C3—C10—C11	−27.28 (16)
C2—C3—C4—C5	18.85 (16)	O4—C10—C11—C16	139.12 (12)
O2—C4—C5—C9	−162.55 (9)	C3—C10—C11—C16	−37.08 (17)
C3—C4—C5—C9	15.69 (15)	O4—C10—C11—C12	−35.60 (16)
O2—C4—C5—C19	−43.25 (13)	C3—C10—C11—C12	148.19 (11)
C3—C4—C5—C19	135.00 (11)	C16—C11—C12—C13	0.88 (18)
O2—C4—C5—C6	81.58 (12)	C10—C11—C12—C13	175.69 (11)
C3—C4—C5—C6	−100.17 (12)	C11—C12—C13—C14	−1.2 (2)
C4—C5—C6—C17	−63.20 (12)	C12—C13—C14—C15	0.3 (2)
C9—C5—C6—C17	179.32 (9)	C13—C14—C15—C16	0.8 (2)
C19—C5—C6—C17	58.35 (12)	C14—C15—C16—C11	−1.1 (2)
C4—C5—C6—C18	179.61 (10)	C12—C11—C16—C15	0.22 (19)
C9—C5—C6—C18	62.13 (11)	C10—C11—C16—C15	−174.43 (12)
C19—C5—C6—C18	−58.83 (12)	C4—C5—C19—C20	−54.58 (13)
C4—C5—C6—C7	61.56 (12)	C9—C5—C19—C20	63.43 (13)
C9—C5—C6—C7	−55.92 (11)	C6—C5—C19—C20	−178.26 (10)
C19—C5—C6—C7	−176.89 (9)	C5—C19—C20—C21	125.34 (13)
C17—C6—C7—C8	172.43 (9)	C19—C20—C21—C23	0.8 (2)
C18—C6—C7—C8	−70.84 (12)	C19—C20—C21—C22	179.41 (13)
C5—C6—C7—C8	47.55 (13)	C8—C7—C24—C25	52.17 (12)
C17—C6—C7—C24	39.50 (13)	C6—C7—C24—C25	−175.58 (10)
C18—C6—C7—C24	156.23 (10)	C7—C24—C25—C26	−130.54 (13)
C5—C6—C7—C24	−85.38 (12)	C24—C25—C26—C28	179.04 (12)
C24—C7—C8—C1	87.10 (12)	C24—C25—C26—C27	−0.3 (2)
C6—C7—C8—C1	−47.26 (13)	C9—C1—C29—C30	−70.74 (12)
C9—C1—C8—C7	52.49 (12)	C2—C1—C29—C30	53.51 (12)
C29—C1—C8—C7	173.57 (10)	C8—C1—C29—C30	172.61 (9)
C2—C1—C8—C7	−66.97 (12)	C1—C29—C30—C31	−159.64 (11)
C29—C1—C9—O3	−9.02 (15)	C29—C30—C31—C32	179.38 (11)
C2—C1—C9—O3	−132.11 (11)	C29—C30—C31—C33	−2.82 (19)
C8—C1—C9—O3	110.23 (12)		

### Hydrogen-bond geometry (Å, º)

**Table:**

*D*—H···*A*	*D*—H	H···*A*	*D*···*A*	*D*—H···*A*
O2—H2*O*···O4	1.014 (16)	1.477 (16)	2.4368 (12)	155.7 (15)
